# Non-invasive diagnostic biomarkers for estimating the onset time of permanent cerebral ischemia

**DOI:** 10.1038/jcbfm.2014.155

**Published:** 2014-09-03

**Authors:** Carole Berthet, Lijing Xin, Lara Buscemi, Corinne Benakis, Rolf Gruetter, Lorenz Hirt, Hongxia Lei

**Affiliations:** 1 Department of Clinical Neurosciences, Neurology Service, Centre Hospitalier Universitaire Vaudois, Lausanne, Switzerland; 2 Laboratory for Functional and Metabolic Imaging, Ecole Polytechnique Fédérale de Lausanne, Lausanne, Switzerland; 3 Unit for Research in Schizophrenia, Center for Psychiatric Neuroscience, Department of Psychiatry, Lausanne University Hospital (CHUV), Lausanne, Switzerland; 4 Department of Radiology, University of Geneva, Geneva, Switzerland; 5 Department of Radiology, University of Lausanne, Lausanne, Switzerland; 6 AIT, Center for Biomedical Imaging (CIBM), Institute of the Physics of Biological Systems, Ecole Polytechnique Fédérale de Lausanne, Lausanne, Switzerland

**Keywords:** biomarkers, magnetic resonance spectroscopy, MCAO, mouse, stroke, stroke onset biomarkers

## Abstract

The treatments for ischemic stroke can only be administered in a narrow time-window. However, the ischemia onset time is unknown in ~30% of stroke patients (wake-up strokes). The objective of this study was to determine whether MR spectra of ischemic brains might allow the precise estimation of cerebral ischemia onset time. We modeled ischemic stroke in male ICR-CD1 mice using a permanent middle cerebral artery filament occlusion model with laser Doppler control of the regional cerebral blood flow. Mice were then subjected to repeated MRS measurements of ipsilateral striatum at 14.1 T. A striking initial increase in *γ*-aminobutyric acid (GABA) and no increase in glutamine were observed. A steady decline was observed for taurine (Tau), *N*-acetyl-aspartate (NAA) and similarly for the sum of NAA+Tau+glutamate that mimicked an exponential function. The estimation of the time of onset of permanent ischemia within 6 hours in a blinded experiment with mice showed an accuracy of 33±10 minutes. A plot of GABA, Tau, and neuronal marker concentrations against the ratio of acetate/NAA allowed precise separation of mice whose ischemia onset lay within arbitrarily chosen time-windows. We conclude that ^1^H-MRS has the potential to detect the clinically relevant time of onset of ischemic stroke.

## Introduction

The time of symptom onset is a critical parameter for the management of acute ischemic stroke patients as the therapeutic window is limited to 4.5 hours for thrombolysis,^1^ 6 hours for intraarterial recanalization procedures^2^ and 9 hours for desmoteplase (Desmoteplase in Acute Ischemic Stroke Trial, DIAS).^3^ However, a significant number of patients have an unknown time of onset as the stroke occurred during their sleep, which disqualifies them for thrombolytic treatment. Therefore, knowing the precise onset time of the ischemic insult would help determine whether a given patient can be treated or not.^4,5^

We have previously shown in mice that ^1^H magnetic resonance spectroscopy (^1^H-MRS)^6^ can reliably estimate at a very early time point the severity of cerebral ischemia induced by middle cerebral artery occlusion (MCAO) and distinguish transient ischemic attacks from minor strokes or moderate strokes based on metabolite concentrations in the ipsilateral striatum,^7^ the ischemic core.^8^ In particular, a scatter plot of a combination score of *N*-acetyl-aspartate (NAA), glutamate (Glu), and taurine (Tau) against glutamine (Gln), accurately measured in the infarcted striatal region 3 hours after ischemia onset, allowed the separation of moderate strokes, resulting from 30-minute MCAO, from minor strokes and transient ischemic attacks induced by 10-minute MCAO and shams.^7^

We now applied the same noninvasive approach^7,9, 10, 11^ to a more severe model of stroke, permanent ischemia (MCAO without reperfusion) in mice. Permanent ischemia is a condition observed in stroke patients in need of revascularization.^1, 2, 3,12^ Our aim was to follow the evolution of spectral changes in the ischemic core^13^ after MCAO without reperfusion by examining animals at different time points after ischemia and correlating spectral changes to the progression of ischemic damage. This allows the identification of potential biomarkers, which might be further used to estimate the time of ischemia onset.

## Materials and Methods

### Permanent Middle Cerebral Artery Occlusion in the Mouse

All animal experiments were approved by the Veterinary Office of Canton de Vaud, and were conducted according to the federal and local ethical guidelines, EXPANIM (Expérience sur animaux- SCAV, Service de la consommation et des affaires vétérinaires, Switzerland). Forty male ICR-CD1 mice (20 to 33 g, Charles River, L'Arbresle, France) were housed under standard conditions with unlimited access to food and water. They underwent MCAO without reperfusion (permanent MCAO) using a filament technique as previously described.^14^ Specifically, mice were anesthetized and kept in 1.5% to 2% isoflurane with 30% oxygen and 70% nitrous oxide using a facemask. At 0 hours, permanent ischemia was induced by inserting a silicone-coated nylon filament (diameter: 0.17 mm, Doccol, Redlands, CA, USA) through the left common carotid artery into the internal carotid artery. Throughout surgery and until awakening, the blood flow was monitored with a laser Doppler (Perimed, Stockholm, Sweden) and the rectal temperature of the animal was maintained at 37±0.5°C with a temperature control unit (FHC, Bowdoin, ME, USA). Ischemia induction was considered successful if the blood flow dropped under 20% of the baseline.

Four mice died and were not measured. Four were excluded because of the loss of Doppler monitoring signals during the ischemia period, absence of elevated creatine levels within 1 hour of ischemia onset, or no ischemic lesion detected by T_2_-weighted magnetic resonance imaging at 24 hours (described later). In addition, the sham group, which underwent the same procedure without any artery ligation or suture insertion, was taken from a previous study.^7^

### Magnetic Resonance Instrumentation and Methods

After permanent MCAO, animals were carefully prepared, positioned, and placed in a horizontal bore (inner diameter 26 cm) 14.1 T magnet (Magnex, Oxford, UK).

T_2_-weighted images (fast spin echo imaging, *ETL*=8, effective echo time and repetition time: *TE*_eff_/*TR*=50/5,000 ms, four scans) were acquired to measure the anatomic structure to localize accurately the volumes of interest and also to monitor the development of T_2_-hyperintensive vasogenic edema. After field homogeneity optimization for the target volume, localized ^1^H-MR spectra^11^ were obtained from the ipsilateral striatum (6 to 8 *μ*L volumes of interest) at different time points up to 25 hours after permanent MCAO. To compare with previous transient MCAO studies,^7^ the current ^1^H-MRS studies were carefully arranged to obtain a sufficient population (i.e. *n*⩾5) for the selected time points after permanent MCAO: 1, 3, 8, and 24 hours.

Brain swelling reflects the edema formation after stroke.^15^ Brain swelling was estimated as follows.^11,15^


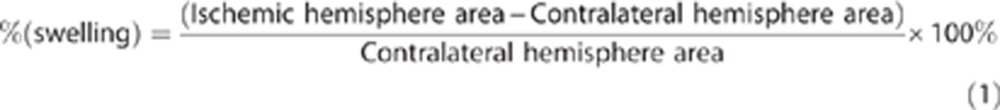


### Data Analysis

The acquired ^1^H-MR spectra were processed as previously described.^7,11^ Specifically, all spectra were frequency corrected, summed, and compensated for eddy currents for further quantification using a home-developed Matlab package. Previous studies showed that brain swelling with a hemispheric volume increase of 20% corresponds to a water content increase of ~4% to 5%.^15^ Such an increase would only contribute negligible error to the estimation of metabolite contents considering the ^1^H-MRS measurement errors, i.e. 10%. Therefore, the MR spectra were quantified using LCModel (Linear Combination MODEL^16^) assuming 80% water content and were scaled accordingly only in the case of brain swelling beyond 20%.^15^ Unaltered patterns of macromolecules were assumed based on previous transient ischemia and results,^7,11^ and thereafter confirmed by evaluating whether the spectrum fit residues were flat.

All statistical tests, i.e. Student's *t*-test and two-way analysis of variance, and fitting procedures were carried on GraphPad Prism 5.0 (GraphPad Software, San Diego, CA, USA). *P* values less than 0.05 were considered significant.

### Ischemia Onset Time Estimation

To evaluate the feasibility of ischemic onset time estimation within time-limited treatment windows,^5^ eighteen permanent ischemia or sham-operated mice were measured by ^1^H-MRS by an observer blinded to the time of ischemia onset and who estimated ischemia onset time based on the acquired metabolite contents. Among these mice, three were excluded because of unsatisfactory blood flow, i.e. >20% of the baseline, which attenuated the induced ischemic damage.^17^ One mouse was measured at two different time points. The ischemia onset time was calculated based on the measured metabolite content and the corresponding best fit. The estimation error for each predicted value was calculated using error propagation, calculated error.

MR measurement times of mice in this blinded experiment were set to 00:00 (hh:mm), both real permanent ischemia onset (PIO) time and the estimated PIO time were expressed relative to the MR measurement time point.

## Results

**Figure 1:**
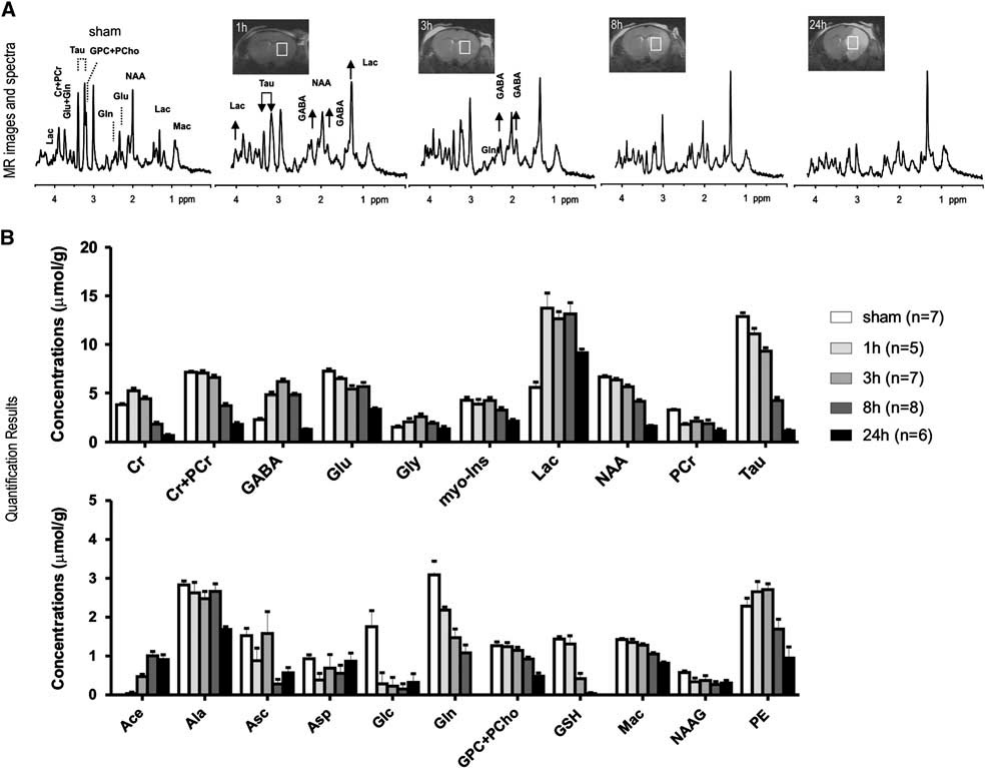
Representative T_2_-weighted magnetic resonance (MR) images and corresponding MR spectra (**A**) from control mice and mice subjected to permanent ischemia at selected time points. White boxes indicate measured regions of interest. Quantification of results is shown in **B**. Sham (white bars), 1 hour (light gray bars), 3 hours (gray bars), 8 hours (dark gray bars) and 24 hours (black bars). Error bars are s.e.m. values. Ace, acetate; Ala, alanine; Asc, ascorbate; Asp, aspartate; Cr, creatine; GABA, *γ*-aminobutyric acid; Glc, glucose; Glu, glutamate; Gln, glutamine; Gly, glycine; GSH, glutathione; GPC, glycerylphosphorylcholine; Mac, macromolecule; myo-Ins, myo-inositol; NAA, *N*-acetyl-aspartate; Lac, lactate; PCho, phosphorylcholine; PCr, phosphocreatine; PE, phosphorylethanolamine; Tau, taurine.

T_2_-weighted magnetic resonance imaging and ^1^H-MRS were carried out on 32 mice subjected to permanent MCAO at various time points. Permanent ischemia induces a T_2_-hyperintensive signal due to edema in the striatum and cortex; visible by 3 hours and thereafter, both the edema size and T_2_-weighted signal intensity increase at 8 hours and 24 hours (Figure 1A). Brain swelling was not observed at 1 hour (−0.1±0.9% of contralateral hemisphere volume), became visible after 3 hours (7.6±2.7%), continued increasing at 8 hours (11.2±2.5%) and increased to 20.0±2.6% at 24 hours, indicating that our metabolite measurements were not significantly influenced by variable water contents due to edema.

Spectral analysis was carried out in the ipsilateral striatum, which was identified in the T_2_-weighted images (Figure 1A, inset images). Upon visual inspection, MR spectra showed highly elevated *γ*-aminobutyric acid (GABA) and more so increased lactate already 1 hour after ischemia, which remained elevated until 8 hours and 24 hours after ischemia, respectively (Figure 1). Elevated lactate concentration was accompanied by diminished glucose (Glc) (Figure 1B).

Furthermore, the increased ratio of creatine to phosphocreatine was visible 1 hour after permanent MCAO (Figure 1, Supplementary Table 1).

In our study, both Tau and myo-inositol were noticeably reduced after permanent ischemia (*P* values<0.05, Figure 1, Supplementary Table 1).

**Figure 2:**
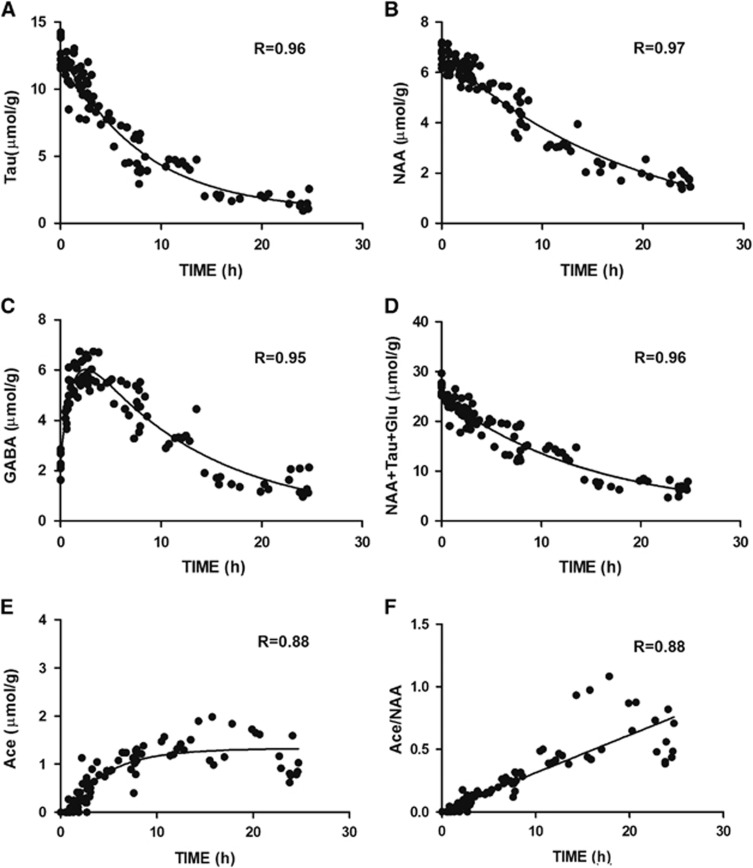
Selected metabolite evolution patterns after permanent middle cerebral artery occlusion, (Tau, **A**); (NAA, **B**); (GABA, **C**); (NAA+Tau+Glu, **D**); (Ace, **E**), and (Ace/NAA, **F**)). Black dots and solid lines represent all data and the corresponding best fit non-linear plots. The resulting *R*-values are reported. Ace, acetate; GABA, *γ*-aminobutyric acid; Glu, glutamate; NAA, *N*-acetyl-aspartate; Tau, taurine.

The presumed neuronal marker NAA decreased gradually over time confirming it as a sensitive neuronal viability marker to ischemia (Figure 1B). The sum of NAA+Tau+Glu decreased gradually after permanent ischemia (Figure 2D) suggesting this score could be used as a marker of neuronal damage after both transient and permanent focal cerebral ischemia.

Acetate (Ace) becomes detectable and rises further after permanent ischemia (Figure 1B), whereas aspartate does not rise. This was further verified at a moderate echo time, i.e., *TE*=40 ms, in which an Ace resonance remained, whereas the GABA signals were minimized because of J-evolution (Supplementary Figure 1).

After ischemia onset, the Glu and Gln decreased, with a steeper decline for Gln (Figure 1B). However, both GABA and glycine increased and remained above those of the sham-operated mice at 8 hours (Figure 1B). Particularly, GABA rose within 1 hour, peaked at ~3 hours, and returned to normal levels by 24 hours (Figure 1B and Supplementary Table 1).

Spectral changes also suggested disturbed cellular proliferation and membrane integrity, shown by the reduction in total choline, phosphorylethanolamine, and macromolecule. Antioxidants (ascorbate and glutathione) also decreased, especially from 8 hours after ischemia onset for glutathione. During the entire study period, we observed no striking alterations in macromolecule patterns.

To identify potential biomarkers that might allow the estimation of the onset time of ischemia, we investigated the evolution over time of metabolite concentrations.

The concentrations of Tau and NAA, as well as the NAA+Tau+Glu composite score declined in a nearly monoexponential fashion (Figures 2A, 2B and 2D, goodness-of-fit, *R*>0.95; Supplementary Table 2) while interestingly, GABA showed a two-phase associated exponential evolution (*R*=0.95) (Figure 2C). In contrast to aspartate, another hydrolytic degradation product of NAA, Ace displayed an exponential increase (Figure 2E, *R*=0.88). When plotting the evolution of Ace/NAA after permanent MCAO, a linear correlation with time after ischemia is clearly seen for the entire time studied (Figure 2F, Pearson *R*=0.88), and even more so for the first 10 hours after permanent MCAO (Pearson *R*=0.95).

**Figure 3:**
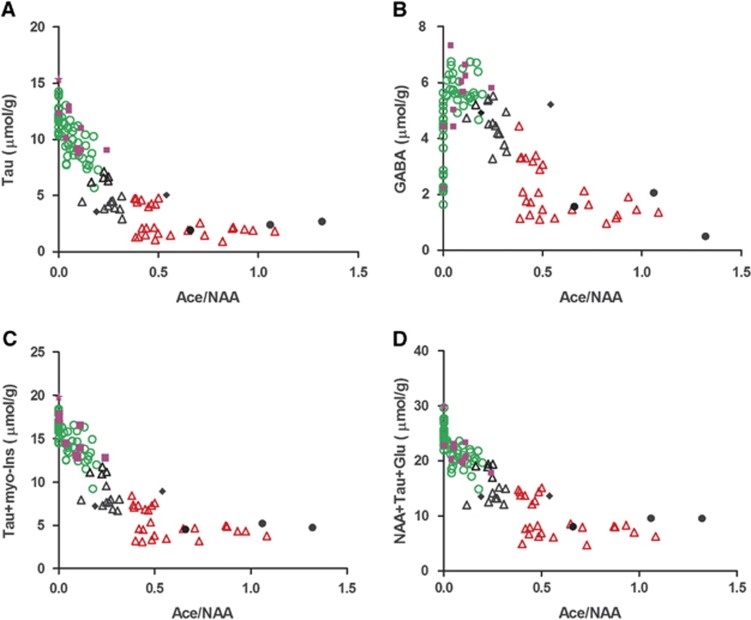
Scatter plots of Tau (**A**), GABA (**B**), putative osmolytes (Tau+myo-Ins, **C**), and neuron-specific metabolites (NAA+Tau+Glu, **D**) against the ratio of Ace/NAA in the following time-windows, 0 to 6 hours (open green circles); 6 to 9 hours (open black triangles); >9 hours (open red triangles). All open shapes therefore represent animals used to establish the plot. The solid purple squares and stars (<6 hours), solid black diamonds (6 to 9 hours), and solid black circles (>9 hours) represent animals of unknown occlusion time to the graph plotter. Ace, acetate; GABA, *γ*-aminobutyric acid; Glu, glutamate; myo-Ins, myo-inositol; NAA, *N*-acetyl-aspartate; Tau, taurine.

In stroke patients, the therapeutic time-windows are 4.5 hours for intravenous thrombolysis, 6 hours for intraarterial recanalization, and 9 hours has been used in recent trials such as DIAS. Arbitrarily and well aware of differences between rodent models and stroke patients, we chose 6 hours and 9 hours after permanent MCAO time points to test whether individual MCAO-operated animals could be classified using their ^1^H-MR spectra within these set time-windows. The Tau and GABA metabolite changes from all permanent MCAO-operated mice were plotted against the ratio Ace/NAA. Mice were then separated into three groups: (1) up to 6 hours (Figure 3, open green circles), the threshold used for intraarterial recanalization procedures, (2) up to 9 hours (open black triangles), the threshold used in DIAS and (3) after 9 hours (open red triangles). A clear separation of the three groups was observed for both the Tau vs Ace/NAA (Figure 3A) and GABA vs Ace/NAA plots (Figure 3B). Interestingly, an equally good separation was achieved using Tau and myo-inositol concentrations (Tau+myo-inositol) and the composite concentration score (NAA+Tau+Glu), shown in Figures 3C and 3D, respectively.

To test the precision of separating mice as either before or after 6 hours, we subjected mice to either permanent MCAO or sham operation and presented them to a masked observer who carried out the ^1^H-MRS analysis. Ten of ^1^H-MR spectral time points (solid purple squares and stars in Figure 3), clearly situated the mice to the within-6-hour (Figure 3, open green circles) group. Among the ten mice, we observed that one data set did not present high GABA or lowered Tau (Figure 3, solid purple stars), similar to neurochemical profiles of healthy control mice (white bars in Figure 1B). We identified this profile as belonging to a sham-operated animal, without permanent MCAO thereby establishing that the technique allows us to determine if there was an ischemic event or not. Three mice showed notably low metabolite contents exhibiting similar neurochemical profiles to mice at 24 hours after permanent MCAO (solid black bars in Figure 1B) and were assigned to the >9 hours group, shown as solid black circles in Figure 3. The remaining two mice were assigned to the 6- to 9- hour group (solid black diamonds in Figure 3). All the estimated MCAO time points from the spectral analysis agreed exactly with the ischemia onset time recorded by the mouse surgeon.

We then determined whether we were able to produce a good estimate of the PIO time based on the metabolite decline curves determined above (Supplementary Table 2).

Table 1 shows our estimations of ischemia onset time, i.e., estimated PIO times, calculated from the decreases of NAA+Tau+Glu, NAA and Tau (Supplementary Table 2) for mice from the blinded experiment (0 to 9 hours) and compares them with the actual permanent MCAO times, real PIO, Table 1. The estimations based on metabolite decays were very precise for the mice measured within 6 hours from the PIO (Table 1), especially using the Tau decline (where differences between real and estimated PIO time range from 5 minutes to 1 hour 9 minutes, 00:41±00:08, mean±s.e.m. values) or NAA+Tau+Glu (where the differences between real and estimated PIO times range from 1 minute to 1 hour 17 minutes, 00:33±00:10). For the two mice measured at later time points (within 9 hours after PIO), the onset time estimates were near 2 hours of the real onset time using NAA+Tau+Glu (2 hours 33 minutes and 1 hour 41 minutes in Table 1).

**Figure 4:**
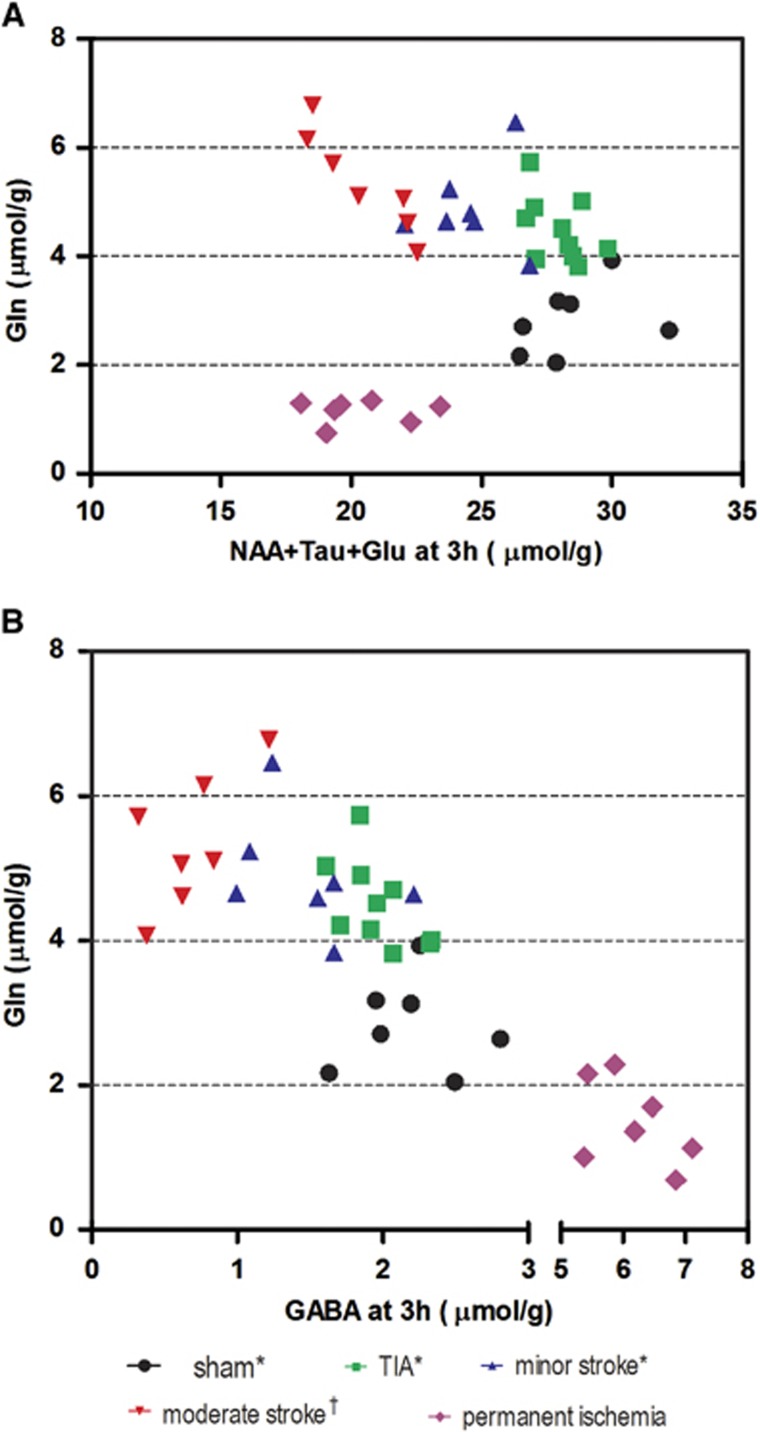
Identification of ischemic subtypes using Gln, NAA+Tau+Glu, (**A**) and GABA (**B**) at 3 hours after middle cerebral artery occlusion. All transient ischemia data and sham data are from Berthet C *et al* (*)^7^ and Lei H *et al* (^†^).^11^ GABA, *γ*-aminobutyric acid; Gln, glutamine; Glu, glutamate; NAA, *N*-acetyl-aspartate; Tau, taurine.

The estimated ischemia onset of the three MCAO-operated mice measured after 9 hours (solid black circles in Figure 4) were all greater than 14 hours, i.e. 16:58±1:06, 24:13±4:07 and 17:22±1:20, respectively.

## Discussion

The present study applied localized ^1^H-MRS to mice subjected to focal cerebral ischemia without reperfusion, providing *in vivo* information on 20 metabolites in the ischemic mouse brain up to 25 hours after ischemia onset and reflecting evolution of the tissue damage. Importantly, we also demonstrate that the metabolite information obtained can be used to provide a good estimate of the ischemia onset time within 6 hours after ischemic insults, extremely relevant if this can be extended to stroke patients and used to determine whether they qualify for intravenous thrombolytic treatment or other therapies that have strict therapeutic windows.

In permanent ischemia, the blood supply is interrupted and not restored. We anticipated a different metabolite evolution pattern and distinct tissue damage compared with transient ischemia^7^ and confirmed this by both the T_2_-weighted images and the MR spectra (Figure 1). T_2_-weighted images (Figure 1) showed that in the permanent ischemia model, the vasogenic edema was not limited to the striatum as in transient ischemia, but also extended to the cortex;^8^ in both regions, T_2_ hyperintensity was just visible 3 hours after ischemia while clearly visible at 8 hours post ischemia. This is different from our previously published observations in transient ischemia in which the T_2_ hyperintensity, if present, does not appear before 8 hours and is only clearly observed one day after minor and moderate strokes.^7^ However, the brain swelling after permanent MCAO reached 8% at 3 hours and kept increasing to 11% at 8 hours, both greater than previously observed in moderate stroke. Even so, this increase in brain swelling implies an increased water content of ~0.7% and therefore only contributes minimally to variability in the metabolic results, i.e., less than 1% underestimation, which becomes negligible compared with experimental error.

The MR spectral patterns in the infarcted striatum in permanent ischemia revealed a very different profile compared with our previously published transient ischemia results. For instance, the decrease of Gln during permanent ischemia (MCAO without reperfusion) was different from that observed after transient-type ischemia, i.e., transient ischemic attack (10 minutes transient ischemia without lesion), minor stroke (10 minutes transient ischemia with lesion) and moderate stroke (30 minutes transient ischemia with lesion).^7^ Furthermore, the sum of NAA+Tau+Glu at 3 hours after permanent ischemia (19.8±1.5 *μ*mol/g) was very similar to that after moderate stroke at 3 hours (20.8±1.8 *μ*mol/g).^7,11^ Thus, plots of Gln against the sum of NAA+Tau+Glu at the 3 hours time point after ischemia allows an excellent separation of all transient ischemia subtypes (Figure 4A), as well as generating clear separation between transient and permanent ischemia at a very early time point.

Of striking interest is the highly elevated GABA in the permanent ischemia MR spectra (Figure 1). This is consistent with energy failure favoring anaerobic GABA synthesis as seen in postmortem tissue.^18, 19, 20, 21, 22, 23, 24^ GABA rises were not detected after transient ischemia.^7,11^ This is most likely due to the interruption of oxygen supply in permanent ischemia. Highly elevated GABA after permanent ischemia has been reported in focal and global ischemia studies using *ex vivo* analysis^25^ but not yet reported by direct *in vivo* measurement.

The combined observation of elevated GABA and decreased Gln (Figure 2) is distinct from that observed in transient ischemia subtypes;^7^ therefore, these changes are potential markers for permanent ischemia in stroke conditions. A plot of Gln against GABA at 3 hours presents a well-defined separation of permanent ischemia from all transient ischemia subtypes (Figure 4B), making *GABA* a new biomarker for identifying non-perfused ischemia from re-perfused subtypes.

To our knowledge, comparing the permanent ischemia results here with our previously published transient ischemia results provides the first comparison of metabolite changes between permanent ischemic insults and different severities of transient ischemic insults, ranging from transient ischemic attacks to moderate strokes.

Shortly after permanent MCAO, i.e., at 1 hour (Figure 1 and Supplementary Table 1), highly elevated creatine and reduced phosphocreatine are observed, and signifies energy failure in global ischemia^26^ and hypoglycemia.^27^ A sudden change in relative ion concentrations after ischemia would explain the observed osmoregulation dysfunction,^25^ for example, the significant reduction of Tau and myo-inositol, described in Figure 1 and Supplementary Table 1.

The decreased Gln and Glu concentrations after permanent MCAO most likely reflect cell death with loss of membrane integrity, as well as energy deficits,^28^ both of which might impair neurotransmission, and are similar to published *ex vivo* results.^25^ The Gln reduction at the beginning of permanent MCAO implies that astrocytes are not able to convert the extracellular accumulated Glu to Gln due to reduced oxygen supply in the ischemic core. This contrasts to the Gln increase seen in transient ischemia where astrocytes are able to carry out the conversion when blood flow is restored.

Moreover, the NAA+Tau+Glu score, identified as a marker of irreversibly damaged tissue in transient ischemia,^7^ remains valid in permanent ischemia. If translated to clinics, this evaluation of irreversible damage could be useful for patient management and patient selection in future neuroprotection trials. Finally, the accumulation of lactate along with the very low glucose content observed shortly after permanent MCAO and which remain at similar levels thereafter, suggests anaerobic glycolysis occurring with reduced oxygen supply.^7^

**Figure 5:**
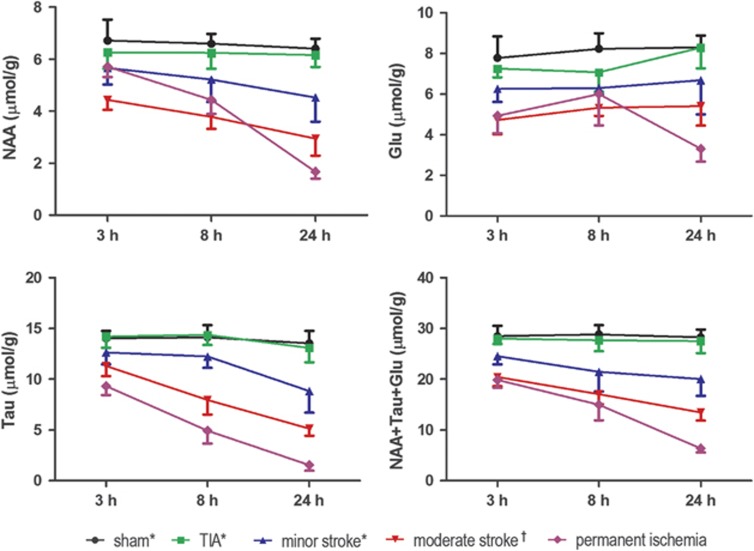
Evolution of neuronal metabolites 3 hours, 8 hours and 24 hours after permanent ischemia was compared with transient ischemia, i.e., transient ischemic attack (TIA), minor stroke and moderate stroke, from Berthet C *et al* (*)^7^ and from Lei H *et al* (^†^).^11^ Glu, glutamate; NAA, N-acetyl-aspartate; Tau, taurine.

Interestingly, 3 hours after ischemia onset, the NAA concentration in permanent ischemia was reduced^29,30^ and similar to that found in minor stroke (Figure 5), whereas at 8 hours after MCAO, the NAA concentration in permanent ischemia was lower than in minor stroke but not as low as in moderate stroke.^7^ The faster NAA decrease seen in transient ischemia, 8 hours after ischemia onset, suggests that there is possible secondary damage occurring after the restoration of blood flow in transient ischemia.^31,32^ Reperfusion therefore induces damage at early time points, but after 1 day, the damage induced by long-lasting blood supply deficit in permanent ischemia likely dominates the transient detrimental effects of reperfusion.

NAA and Tau gradually declined in a monoexponential fashion. This is likely caused by the lack of restoration of cerebral blood flow. Previous studies have shown that there is a decline in osmolytes, an increase in intracellular water content, and accumulation of sodium in the ischemic brain tissue.^25,33, 34, 35, 36^ Interestingly, sodium magnetic resonance imaging in rats has been proposed as a way to estimate stroke onset time.^36^ We hypothesized that the evolution of these metabolites, e.g., NAA and Tau, after permanent occlusion might enable evaluation of the ischemia onset time.

In the two decay curves (Figure 2), Tau showed a steeper decrease than NAA after permanent ischemia, even more so within 6 hours (Supplementary Table 2). Therefore, we reasoned that Tau decline would provide superior prediction power of onset time in this period. Calculation of onset time based on the NAA concentration alone was, as expected, less precise than Tau alone (Table 1). When testing mice up to 6 hours after ischemia onset, we noticed that the estimated onset time from the modeled declines of the metabolite score NAA+Tau+Glu lay within a satisfactory range of the real onset time thereby providing good estimation (Table 1).

Overall, when Tau concentration declines are large, the predicted ischemia onset times are very accurate; in contrast to Tau declines that are smaller than the ^1^H-MRS measurement error where prediction is less accurate. However, it should be pointed out that small declines only happen shortly after permanent ischemia indicative of being very close to the onset time. Furthermore, the combination score of NAA, Tau and Glu would improve the estimation precision at very short times after permanent MCAO (Table 1).

Notably, the linear increase of Ace/NAA (Figure 2F) after permanent MCAO is reported for the first time as well as GABA, as potential biomarkers for permanent ischemia. Even though the reason of Ace increase after permanent MCAO remains unclear but might be caused by NAA degradation,^37^ the combination of Ace/NAA with other metabolite(s) such as GABA, NAA+Glu+Tau (Figure 4) can situate individual animals into potential time-frame windows allowing to evaluate therapeutic interventions in animal models of stroke. Importantly, combined with highly improved ^1^H-MRS techniques in clinical platforms,^38,39^ ^1^H-MRS could become applicable for future clinical studies on stroke patients.

## Conclusion

We conclude that ^1^H-MRS is a powerful noninvasive tool for the estimation of the onset time of ischemia in mice. The time of onset is critical and an MRS based estimation of onset time could be a criterion to decide whether thrombolytic treatment should be used.

## Acknowledgement

We thank Dr Melanie Price for critical evaluation and help with editing the manuscript.

## Figures and Tables

**Table 1:** Summary of PIO time predictions

*NAA*			*Tau*			*NAA+Tau+Glu*			
*Conc (*μ*mol/g)*	*Est PIO time (hh:mm)*	*Delta (hh:mm)*	*Conc (*μ*mol/g)*	*Est PIO time (hh:mm)*	*Delta (hh:mm)*	*Conc (*μ*mol/g)*	*Est PIO time (hh:mm)*	*Delta (hh:mm)*	*Real PIO time (hh:mm)*
6.1	−01:57±00:27	00:56	11.0	−01:19±00:12	00:18	23.4	−01:21±00:19	00:20	−01:01^a^
5.6	−03:18±00:42	02:06	13.0	00:00±00:09	01:12	23.2	−01:29±00:20	00:17	−01:12
4.9	−05:39±01:12	04:30	12.6	−00:08±00:09	01:01	22.5	−01:56±00:24	00:47	−01:09
5.3	−04:14±00:54	02:46	12.4	−00:19±00:10	01:09	22.9	−01:40±00:22	00:12	−01:28
6.0	−02:08±00:29	00:08	10.1	−02:03±00:16	00:13	20.3	−03:25±00:38	01:09	−02:16
5.6	−03:32±00:45	00:55	8.8	−03:20±00:23	00:43	19.7	−03:54±00:43	01:17	−02:37
5.3	−04:50±00:54	01:45	9.1	−03:00±00:21	00:05	20.8	−03:03±00:34	00:02	−03:05
4.8	−06:07±01:17	02:34	9.4	−02:47±00:20	00:46	19.0	−04:28±00:49	00:55	−03:33
5.9	−02:35±00:34	01:05	9.2	−02:53±00:21	00:47	20.0	−03:39±00:40	00:01	−03:40^a^
4.8	−06:10±01:17	01:07	3.6	−12:10±01:36	04:53	13.5	−09:50±01:51	02:33	−07:17
2.6	−16:32±03:42	08:34	5.0	−08:36±01:01	00:38	13.6	−09:39±01:49	01:41	−07:58

All MR measurement times of mice used in the permanent ischemia onset (PIO) prediction study were set to 00:00 (hh:mm), as described in Methods. Each table row contained one set of data analysis based on N-acetyl-aspartate (NAA), taurine (Tau), and NAA+Tau+Glu from each blinded measurement. Glu, glutamate; Conc, measured metabolite contents; Est PIO time, estimation of ischemia onset times using NAA, Tau, and NAA+Tau+Glu decay curves (Figure 3, Supplementary Table 2) and their corresponding calculated errors using error propagation (see in Methods); Real PIO time, real occlusion time; and Delta, absolute value of the difference between est PIO and real PIO.

a Indicated that one mouse was measured at two time points, as in Methods.
