# Supplementary material for: Non-invasive diagnostic biomarkers for estimating the onset time of permanent cerebral ischemia
Source: J Cereb Blood Flow Metab. 2014 Sep 3;34(11):1848–55. doi: 10.1038/jcbfm.2014.155 (PMC4269763; doi:10.1038/jcbfm.2014.155)
Supplement: Supplementary Information [file jcbfm2014155x1.doc]

**Supplementary Figure 1**


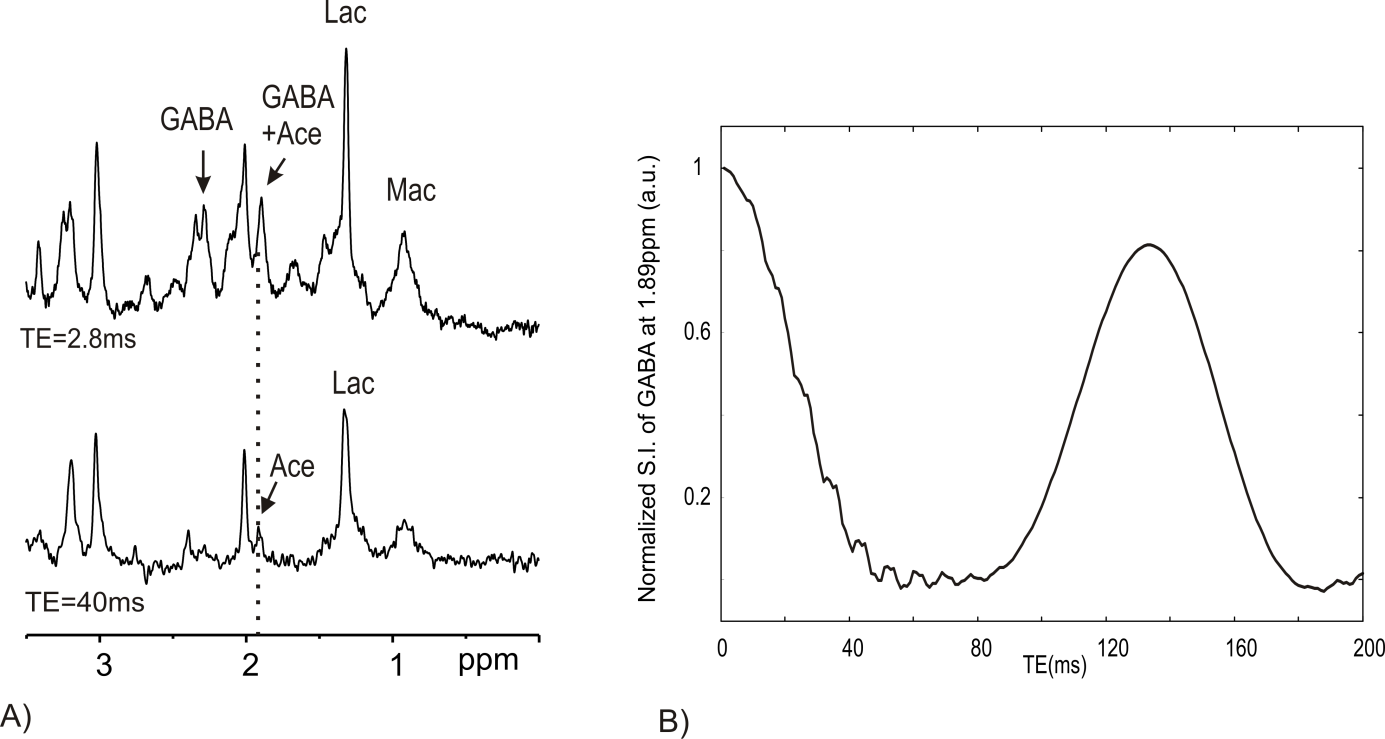


^1^H MRS detection of acetate (Ace) after permanent MCAO. Signal intensity of one GABA resonance at 1.89 ppm (A) was minimized at moderate echo times (TEs), in the range of 40-80ms (B).

**Supplementary Table 1.** Summary of metabolite changes between sham and selected time points, 1, 3, 8 and 24 h after permanent MCAO (pMCAO), and their potential evolution patterns.

| **PUTATIVE FUNCTION** | | **Metabolite** | **Metabolite changes after pMCAO** | | | | **Evolution pattern after pMCAO** |
| --- | --- | --- | --- | --- | --- | --- | --- |
|  |  |  | **1h (n=5)** | **3h (n=7)** | **8h (n=8)** | **24h (n=6)** |  |
| **Energy metabolism** | **Metabolic substrates** | **Glc** | ↓* | ↓*  - | ↓*  -  - | ↓*  -  -  - | Decreased |
|  |  | **Lac** | ↑**** | ↑****  ↓ | ↑****  ↓  ↓ | ↑****  ↓††  ↓‡‡  ↓§§ | Increased |
|  |  | **Ace** | ↑ | ↑****  ↑†††† | ↑****  ↑††††  ↑‡‡ | ↑***  ↑†††  ↑‡  ↓ | Detectable, two-phase evolution |
|  | **Carriers** | **Cr** | ↑** | ↑*  ↓ | ↓****  ↓††††  ↓‡‡‡‡ | ↓****  ↓††††  ↓‡‡‡‡  ↓§§§ | Two-phase associate decay |
|  |  | **PCr** | ↓**** | ↓*  - | ↓**  -  - | ↓****  ↓†  ↓#  ↓ | Decreased |
|  |  | **tCr** | - | ↓  ↓ | ↓****  ↓††††  ↓‡‡‡‡ | ↓****  ↓††††  ↓‡‡‡‡  ↓§§§§ | Mono-exponential decay |
| **Neurotransmitter** | **Inhibitory** | ***GABA*** | *↑**** | ↑****  *↑††* | ↑****  -  *↓‡‡* | ↓****  ↓††††  ↓‡‡‡‡  *↓§§§§* | Two-phase associate decay |
|  |  | **Gly** | ↑ | ↑*  ↑ | ↑  ↓  ↓ | -  ↓  ↓‡  ↓§§ | Two-phase associated evolution |
|  | **Excitatory** | **Glu** | ↓* | ↓**  ↓* | ↓**  ↓  - | ↓****  ↓††††  ↓‡‡‡  ↓§§§ | Decreased |
|  |  | **Gln** | ↓**** | ↓***  ↓† | ↓****  ↓†††  ↓ | ↓****  ↓††††  ↓‡‡‡  ↓§§ | Decreased |
|  | **Neuromodulator** | **Asp** | ↓* | -  - | -  -  - | -  -  -  - | Not increased |
|  |  | **NAAG** | ↓ | ↓  - | ↓**  -  - | ↓*  -  -  - | Decreased |
| **Cellular proliferation/Membrane integrity** | | **NAA** | ↓ | ↓***  ↓† | ↓****  ↓††††  ↓‡‡‡‡ | ↓****  ↓††††  ↓‡‡‡‡  ↓§§§ | Mono-exponential decay |
|  |  | **tCho** | - | ↓  ↓ | ↓**  ↓†  ↓‡ | ↓****  ↓†††  ↓‡‡‡  ↓§§ | Mono-exponential decay |
|  |  | **PE** | ↑ | ↑  - | ↓  ↓†  ↓ | ↓****  ↓††  ↓‡  ↓§§§§ | Two-phase associate decay |
|  |  | **Mac** | ↓ | ↓**  ↓ | ↓****  ↓†  ↓‡‡‡ | ↓****  ↓††  ↓‡‡‡‡  ↓§§§ | Mono-exponential decay |
| **Osmoregulators** | | ***Tau*** | *↓** | ↓****  *↓†* | ↓****  ↓††††  *↓‡‡‡‡* | ↓****  ↓††††  ↓‡‡‡‡  *↓§§§§* | Mono-exponential decay |
|  |  | ***myo*-Ins** | - | -  - | ↓*  ↓  ↓‡ | ↓****  ↓†  ↓‡‡‡‡  ↓§§ | Mono-exponential decay |
| **Antioxidant** | | **GSH** | ↓ | ↓***  ↓†† | ↓****  ↓††  ↓‡ | ↓****  ↓††  ↓‡  ↓ | Decreased |
|  |  | **Asc** | - | -  - | ↓***  ↓  ↓ | ↓**  ↓  ↓  - | No change, then decreased |

The metabolites are classified according to their putative functions despite other potential roles, e.g. taurine as osmolyte. Arrows indicate changes (“↑” for increased and “↓” for decreased, unpaired student t test) when comparing the corresponding time point with healthy controls (row 1), 1h after pMCAO (row 2), 3h after pMCAO (row 3) and 8h after pMCAO (row 4). Significant differences are labeled using the following symbols, “*”, “†”, “‡” and “§”, corresponding to p-values 0.05, 0.01, 0.001 and 0.0001 respectively. “-”: no apparent change. Among the metabolites, GABA and Tau (in italics) were highly significantly different from others at the same time point.

**Supplementary Table 2** Fit results of NAA, Tau and NAA+Glu+Tau.

|  | **NAA** | **Tau** | **NAA+Tau+Glu** |
| --- | --- | --- | --- |
| **Y0 (μmol/g)** | 6.8±0.1 | 12.8±0.2 | 25.6±0.4 |
| **Plateau (μmol/g)** | -0.6±0.8 | 0.9±0.4 | 2.9±1.6 |
| **K (1/h)** | 0.052±0.009 | 0.123±0.012 | 0.078±0.012 |
| **Half time (h)** | 13.3 | 5.6 | 8.9 |

Fit model: Y= (Y0-Plateau)*exp(-K*X)+Plateau. Y0: concentration at 0 h (μmol/g); Plateau: concentration at infinite times (μmol/g); K: rate constant (1/h); X: time (h); Y: concentration at X h (μmol/g).
